# Language, culture and international exchange of virtual patients

**DOI:** 10.1186/1472-6920-13-21

**Published:** 2013-02-11

**Authors:** Valentin Muntean, Tudor Calinici, Stefan Tigan, Uno GH Fors

**Affiliations:** 1 University of Medicine and Pharmacy “Iuliu Hatieganu”, Cluj-Napoca, Romania; 2 Department of Computer and Systems Science, Sweden, Stockholm University, Stockholm, Sweden

**Keywords:** Virtual patients, Computer simulation, Language, Cultural competency, International educational exchange, Medical education

## Abstract

**Background:**

Language and cultural differences could be a limiting factor for the international exchange of Virtual Patients (VPs), especially for small countries and languages of limited circulation. Our research evaluated whether it would be feasible to develop a VP based educational program in our Romanian institution, with cases in English and developed in a non-Romanian setting.

**Method:**

The participants in the research comprised 4^th^ year Romanian medical students from the Faculty of Medicine in Cluj-Napoca, Romania, with previous training exclusively in Romanian, good English proficiency and no experience with VPs. The students worked on eight VPs in two identical versions, Romanian and English. The first group (2010) of 136 students worked with four VPs developed in Cluj and the second group (2011) of 144 students with four VPs originally developed at an US University. Every student was randomly assigned two different VPs, one in Romanian and another in English. Student activity throughout the case, the diagnosis, therapeutic plan and diagnosis justification were recorded. We also compared student performance on the two VPs versions, Romanian and English and the student performance on the two sets of cases, originally developed in Romania, respectively USA.

**Results:**

We found no significant differences between the students’ performance on the Romanian vs. English version of VPs. Regarding the students’ performance on the two sets of cases, in those originally developed in Romania, respectively in the USA, we found a number of statistically significant differences in the students’ activity through the cases. There were no statistically significant differences in the students’ ability to reach the correct diagnosis and therapeutic plan.

**Conclusion:**

The development of our program with VPs in English would be feasible, cost-effective and in accordance with the globalization of medical education.

## Background

Virtual Patients (VPs) are learning systems designed to simulate encounters between a patient and a healthcare professional [1]. VPs may be used throughout the medical curriculum including pre-clinical courses [2]. Virtual Patients are commonly recommended for teaching clinical reasoning and clinical decision making, but have also been used for teaching basic communication skills with patients [3-5]. There are also suggestions for the use of VPs to emphasize socio-cultural aspects and cultural differences as they pertain to healthcare education [6,7].

Developing a virtual patient (VPs) program at a single institution, to comprehensively cover a curriculum, is virtually impossible. Quality computer-assisted instruction materials are time and labour intensive to develop, and therefore expensive. The development and maintenance of virtual patients in medical education through a collaborative multi-institutional authoring might be the best solution for most medical schools [8]. The option to use VPs from other universities may therefore be appealing, but these may only be available in English, German or French. In addition, even if most medical students in Europe can read English, cases developed in USA or UK might display conditions and other cultural issues that can impede the educational value [6]. Moreover, if in many cases repurposing was efficient when compared to the larger amount of time needed for *de novo* VPs [9,10], translation of VP’s in the native learners language is still resource consuming and a limiting factor in their use and international exchange.

In the “Iuliu Hatieganu” University of Medicine and Pharmacy in Cluj-Napoca, Romania, English is mandatory for students enrolled in all programs. In a pilot study, comparing the performance of Romanian students on English and Romanian VPs, we found a better diagnosis and treatment plan in Romanian versus English versions [6]. The present research tried to find out if using VP’s in English would be a viable option for developing a VPs program in our institution.

## Methods

The participants in the research study were 4^th^ year Romanian medical students from the Faculty of Medicine in Cluj-Napoca, Romania, enrolled in the optional course: “Methods of teaching and evaluation for medical students” for two consecutive years (2010 and 2011). The students’ previous training was exclusively in Romanian and none of them had worked with VPs before. A requisite for participation in the study was a good English proficiency, equal to or better than the B1 level. The participation in the study was optional and the identity of the students remained unknown to the researchers throughout the study. The research design, including ethical issues, was approved by the Dean, the Curriculum Office and the Ethics Committee of the Cluj-Napoca Faculty of Medicine.

**Figure 1:**
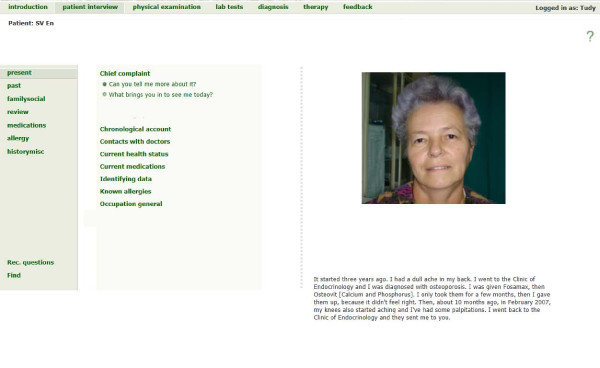
Silvia Vlasa, ENG.

**Figure 2:**
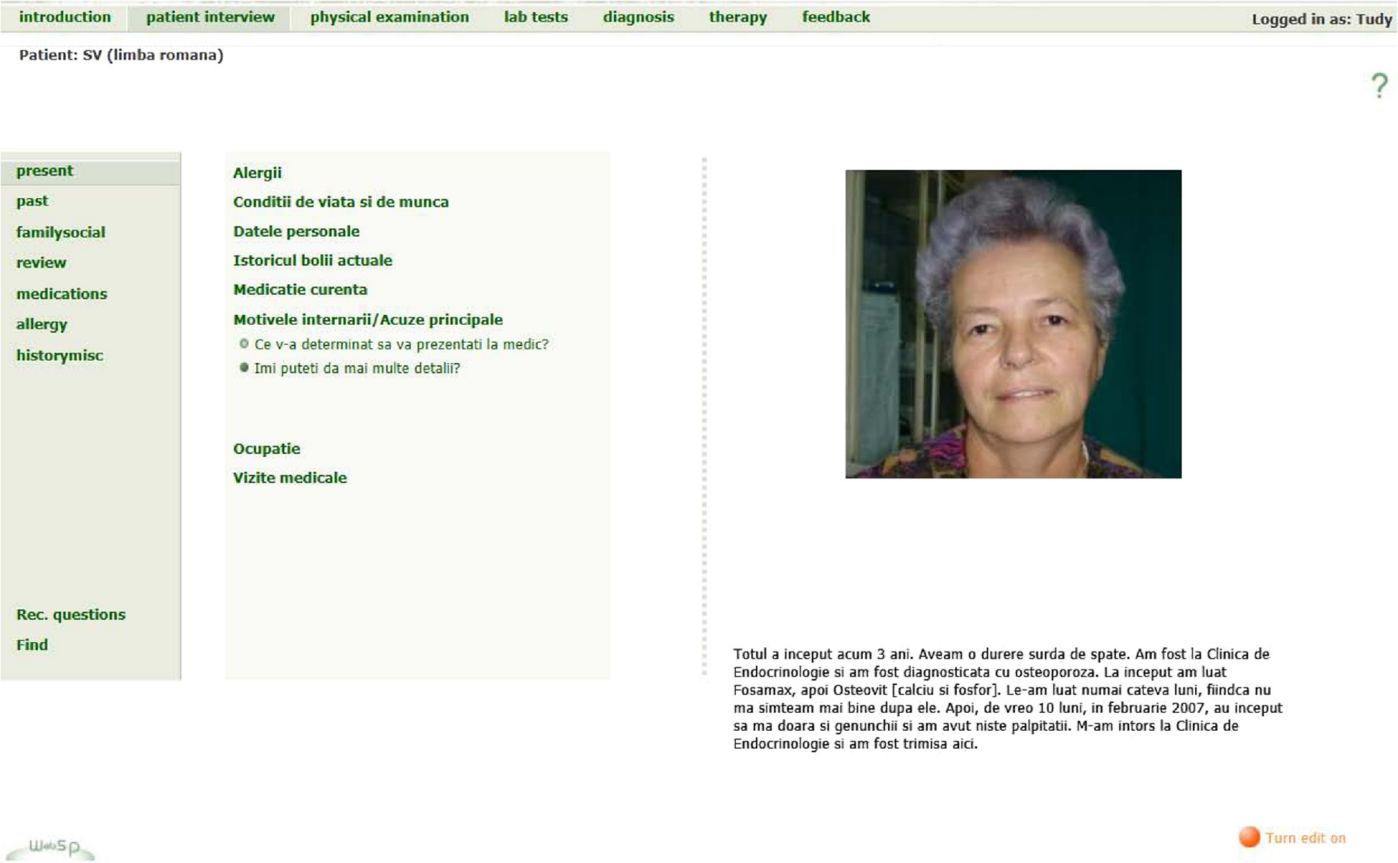
Silvia Vlasa, ROM.

The first group of students (2010) worked on VPs developed by the academic staff in the Internal Medicine Department of Cluj-Napoca–Figures 1 and 2. Four cases were initially developed in Romanian, and then translated into English by the author. The content and details of the versions were identical, and the translation into English was reviewed by a native English speaker. The total repurposing time for the four cases (translation into English, editing, review and final check) was 30, 25, 33 and respectively 36 hours (mean 31 hours). For the two identical versions, Romanian and English, of each VP, the same checklist, developed by the case author, was used. Cases and checklists were developed based on practice guidelines for diagnostic and therapy, provided by the Internal Medicine Department of Cluj-Napoca. Every case was peer reviewed in terms of content, design and media by two teachers from the Commission for Students. The cases had been used for student training and evaluation for more than two years and rated in student evaluation as “very good” and “of medium difficulty”. The software used was the Web-based Simulation of Patients (Web-SP) system, originating from the Karolinska Institutet, Stockholm, Sweden [11,12].

136 medical students were enrolled in the 2010 course and all volunteered to participate in the study. Every student was given a name code (anonymous) and was randomly assigned two different cases, one in English and one in Romanian. The students were instructed to work with the VP cases as if they were real patients, and ask illness history questions, perform physical exams, order lab/imaging tests as well as suggesting correct diagnoses and therapy with justifications. Access to the cases was given for a seven day period and the students logged on through the Internet in their homes. The cases were supposed to be solved in the two hours allocated per week for independent activity in the optional course “Methods of teaching and evaluation for medical students “. The software provided a help function for queries and technical support was offered by the Information Technology Department of Cluj-Napoca University of Medicine. The students were given no recommendation on which language, Romanian or English, they should use for answering the free text questions regarding diagnosis, therapy and justifications.

Of the 136 students enrolled in the first study, five did not complete both cases and were excluded from the final analysis. The 131 remaining students finalized 262 interactions, half in Romanian and half in English. Because of the randomization of cases and the exclusion from the study of the five students who did not complete both cases, in the end the number of interactions available for the 8 VPs varied between 25 and 40. For each of the 8 VPs versions (4 VPs in Romanian and 4 VPs in English), we selected the chronologically first 25 interactions (history questions, exams, lab/imaging tests etc.) per case (8x25=200 interactions) for statistical analysis. The following criteria were analyzed: Time (minutes) to complete the case; Number of history questions asked; Number of physical examination procedures; Number of laboratory or imaging tests ordered; Diagnosis (Good, Incomplete, or Wrong – numeric correspondence 10, 8 respectively 4); Therapeutic plan (Good, Incomplete, or Wrong – numeric correspondence 10, 8 respectively 4); Student answer (in English or Romanian); Number of words in diagnosis justification. The diagnostic, therapeutic plan and diagnosis justification were analyzed and graded using checklists developed by the case authors and the academic staff of the Department of Internal Medicine.

The second group of students (2011) worked on four VPs originally developed in the USA, with the Web-SP software. In the student evaluation the cases were of medium difficulty and very good, in terms of content, design and media. The translation into Romanian was made by teachers from the Internal Medicine Department. For the two identical versions, English and Romanian, of each VP, the same checklist, developed by the case translator, was used. The content of the versions was identical, and the translation was reviewed by a native English speaker. The total repurposing time for the four cases (translation into Romanian, editing, review and final check) was 27, 23, 24 and respectively 30 hours (mean 26 hours).

Four out of the 144 students enrolled in the second (2011) study did not complete both cases and were excluded from the final analysis. The 140 remaining students finalized 280 cases, half in Romanian and half in English. Because of the randomization of cases and the exclusion f from the study of the four students who did not complete both cases, in the end the number of interactions available for the 8 VPs varied between 26 and 44. For each of the 8 VPs versions (4 VPs in Romanian and 4 VPs in English), we selected the chronologically first 25 interactions (8x25=200 interactions) for statistical analysis.

Statistical analysis was made in SPSS version 16. For both the 2010 and 2011 groups of students we compared the pairs of answers (the performance on the cases) of each student for the two VPs, one in Romanian and another in English that she/he had to complete. Because all the quantitative data series did not have normal distribution, we considered the results of nonparametric tests for statistical analysis.

The comparative analysis of the two sets of cases, the 2010 VPs, developed in Romania by teachers in our Faculty and the 2011 VPs, developed in the USA) was performed with the nonparametric Mann Whitney test (data series with no normal distribution). For the two sets of cases we compared the 2010 Romanian versus 2011 Romanian, 2010 English versus 2011 English and 2010 Romanian + English versus 2011 Romanian + English.

## Results

In the first sub-study, with VPs developed in Cluj (the 2010 group), we found no important differences between the student performance in Romanian when compared with the performance in the English version of VPs, see Table 1. The only statistical significant difference between the pairs Romanian / English was the therapeutic plan. The variance of data is large for all parameters studied, showing considerable difference among student activity through the cases. Student answers, diagnosis, therapeutic plan and diagnosis justification, were in Romanian 171 times and in English 29 times, all for VPs in English.

**Table 1:** Comparison of student performance on Romanian versions (ROM) versus English versions (ENG) of VPs

		**Time / case (min)**		**Number questions history**		**Number questions physical**		**Number questions lab. & imag.**		**Diagnosis (good – 10 incomplete – 8 wrong – 4)**		**Therapeutic plan (good – 10 incomplete – 8 wrong – 4)**		**Number of words in diagnosis justification**	
**VPs LANGUAGE**		**RO**	**ENG**	**RO**	**ENG**	**RO**	**ENG**	**RO**	**ENG**	**RO**	**ENG**	**RO**	**ENG**	**RO**	**ENG**
2010 Cluj VPs	MEAN	47.00	47.00	49.00	51.00	25.00	22.00	23.00	24.00	7.04	7.32	6.08	6.62	27.00	25.00
	SD	25.69	10.10	33.11	35.55	28.93	28.64	14.35	13.86	2.19	2.11	2.18	2.23	20.64	21.35
	p-value (two-tail)	0.9662		0.6271		0.4088		0.7404		0.5679		**0.0768**		0.1977	
2011 USA VPs	MEAN	42.00	46.00	84.00	83.00	28.00	28.00	22.00	25.00	6.96	7.30	6.46	6.42	33.00	28.00
	SD	33.02	43.76	61.90	59.77	14.53	16.25	19.08	35.85	2.35	2.42	2.05	2.06	27.33	17.26
	p-value (two-tail)	0,2297		0,8524		0,7682		0,4386		0,2716		0,8789		**0,0744**	

The VPs developed in Cluj (2010 Cluj VPs) and the VPs developed in the USA (2011 USA VPs).

For the second, the 2011 group, working on American VPs, we also found no important difference when comparing the Romanian version versus the English version of VPs, see Table 1. The single data set with a statistically significant difference between the Romanian / English pairs was for the number of words in diagnosis justification. The variance of data (student activity through the cases) for all parameters studied is even larger than for the 2010 group. Again, most student answers were in Romanian, 174 times, and only 26 times in English, all for VPs in English.

Regarding the comparison between the two sets of cases, those with authors in Cluj (2010 group), and those developed in the USA (2011 group), we found a number of statistically significant differences in student activity through the cases – Table 2. The 2010 group of students spent more time on the cases, asked for a lesser number of history questions and physical queries and more laboratory and imagistic information and used less words in diagnosis justification when compared with the 2011 group. There were no statistical differences in the diagnostic and therapeutic plan on the two sets of cases. The lack of significant statistical differences in the students’ ability to reach the correct diagnosis and treatment plan persisted when we separately compared the Romanian and English versions of the Cluj versus American VPs.

**Table 2:** Comparison of student performance on VPs developed in Cluj (2010 - Cluj) and the VPs developed in the USA (2011 - USA)

			**Time / case (min)**	**Number questions history**	**Number questions physical**	**Number questions lab. & imag.**	**Diagnosis (good – 10 incomplete – 8 wrong – 4)**	**Therapeutic plan (good – 10 incomplete – 8 wrong – 4)**	**Mumber of words in diagnosis justification**
ROM + ENG	2010 - Cluj	Mean +/− SD	47 +/− 28	50 +/− 34	23 +/− 28	24 +/− 14	7.23 +/− 2.14	6.35 +/− 2.21	26 +/− 20
	2011 - USA	Mean +/− SD	44 +/− 38	84 +/− 60	28 +/− 15	24 +/− 28	7.13 +/− 2.38	6.44 +/− 2.04	31 +/− 22
	2010 - Cluj versus 2011 - USA	p-value (2-tailed)	**.011**	**.000**	**.000**	**.008**	.987	.758	**.021**
ROM	2010 - Cluj	Mean +/− SD	47+/−26	49+/−33	25+/−29	23+/−14	7.04+/−2.19	6.08+/−2.18	27+/−21
	2011 – USA	Mean +/− SD	42+/−33	84+/−62	28+/−15	22+/−19	6,96+/−2.35	6.46+/−2.05	33+/−27
	2010 -Cluj versus 2011 - USA	p-value (2-tailed)	**.039**	**.000**	**.000**	.134	.420	.665	**.038**
ENG	2010 - Cluj	Mean +/− SD	47 +/− 10	51 +/− 36	22 +/− 29	24 +/− 14	7.32 +/− 2.11	6.62 +/− 2.23	25 +/− 21
	2011 - USA	Mean +/− SD	46 +/− 44	83 +/− 60	28 +/− 16	25 +/− 36	7.30 +/− 2.42	6.42 +/− 2.06	28 +/− 17
	2010 -Cluj versus 2011 - USA	p-value (2-tailed)	**.035**	**.000**	**.000**	.087	.480	.310	.112

Romanian and English versions (ROM + ENG), Romanian versions (ROM) and English versions (ENG) of VPs. Nonparametric Mann–Whitney test.

## Discussion

High quality computer-assisted instruction materials are time and labour intensive to develop, and therefore expensive [13]. One interesting learning tool for medical education is Virtual Patients (VPs), where the learner effectively might train for clinical reasoning and critical thinking [1,5,14]. VPs are also be used for assessment [15].

As partners in the three-year EU-funded project, called ‘eViP, we started to repurpose some Cluj Faculty of Medicine VPs in the Web-based Simulation of Patients (Web-SP) system from Sweden. The repurposing language was Romanian and English, the latter necessary for our undergraduate program with teaching in English. Furthermore, we obtained VPs in English (medicine and dentistry) for repurposing into Romanian from Karolinska University. The VPs were initially used in the PBL course during the first two preclinical years and later, in the clinical years, as "blended learning". The feedback received from students was positive. Student evaluation rated VPs funny, engaging and informative, offering great value in learning and evaluation.

The design of the cases edited in the Web-based Simulation of Patients (Web-SP) was 'narrative', not only best suited for history-taking and communication skills [16], including diagnostic ability [4], but also for learning and the assessment of clinical reasoning [5,17,18]. The criteria used for the assessment of the students’ activity on the cases were those offered by the software, namely ordering correct illness history, physical exam, lab/imaging tests, and suggesting correct diagnosis, therapy and justifications of those. The students’ ability to obtain the correct diagnosis and treatment was assessed and graded using checklists developed by the case authors. The cases selected for this research, from both Cluj and USA, were from the Internal Medicine field and of mean difficulty, chosen by the academic staff from the Department of Internal Medicine in Cluj.

Even if a pre-test was not performed, we presumed that the two groups had a similar level of knowledge and skill. The 2010 and 2011 groups of students had similar background and training. All fourth year medical students participating in the research had previously studied Internal Medicine and none had worked with VPs before. The previous training of the students was exclusively in Romanian and all of them had a good English proficiency (equal to or better than the B1 level).

Good knowledge of English is mandatory for all our students and printed and electronic materials in English, French or German, as recommended readings for lectures, seminars and clerkships are regularly used. That is why, three years ago, when we started with the VPs edited in the Web-SP software during the optional course “Methods of teaching and evaluation for medical students”, VPs in both Romanian and English were utilised. The initial impression was that Romanian students’ performance on Romanian VPs is better than on the English versions (better diagnosis and treatment plan) [6].

However, the current study contradicts the previous supposition. When comparing the students’ activity on the cases and their ability to reach the correct diagnosis and treatment, we found only minor differences between the Romanian and English versions of a certain VP. This statement is true for both cases developed in Cluj as well as for those from the USA. Furthermore, there were no statistical differences on the students ability to reach the correct diagnosis and therapeutic plan between VPs with Cluj authors (2010 - Cluj), and VPs developed in the USA (2011 - USA).

Development of quality VPs takes time and is expensive, with a wide range of production costs, associated with personnel, software or technical infrastructure [13]. This explains why obtaining computer-assisted instruction materials used extensively in medical education is difficult to achieve [8]. The broad use of VPs depends on the transition of the programs from relying on grant funding to financially self-sustaining, through a multi-institutional authoring collaboration [8].

The development and maintenance of virtual patients in medical education through a collaborative process, based on a commons model, was proposed as an attractive solution for supporting complex and costly activities. A virtual patient commons then is one where a particular community creates, adapts, shares, reuses and otherwise makes use of a bank of virtual patient cases, held by and on behalf of that community [1].

Any viable commons is based on standards for the re-purposing and sharing of system components [19]. The development of an open data interoperability standard for virtual patients has been a key component in enabling collaborative approaches. The XML-based "MedBiquitous Virtual Patient Standard" (MVP) describing a common structure for virtual patient content and activities enables virtual patient exchange across systems, modification, and display within conformant player software [20]. The MedBiquitous Virtual Patient standard is now adopted by all the major Virtual Patient systems in Europe and North America [21]. Legal issues, related to patient consent for distribution of multimedia [22] and intellectual property rights, digital copyright issues and licensing/sharing model [23], should be clarified. Other important aspects include specific approaches to metadata, vocabularies, language and cultural norms, all of which make up the commons’ specific profile [1]. The description of different VP designs typology provides a common reference point for all those wishing to report on or study VPs [24]. This metadata will eventually permit the case to be included in larger repositories of virtual patient cases and encourage utilization among schools and collegial sharing [25].

For small countries the language is an important factor that limits participation in a collaborative, multi-institutional authoring program. The VPs available for sharing could be only in languages of large circulation, i.e. English [8,21], French [26] or German [27,28]. As partners in the ‘eViP’ EU-funded project we translated / repurposed cases offered by the Karolinska Institutet. Even if in many cases repurposing was efficient when compared to the larger amount of time needed for *de novo* VPs [21], translation into Romanian took time and effort. In this research, the mean time/case for repurposing (translation, editing, review, and final check) was 31 hours for Romanian to English and 26 hours for English to Romanian.

Besides the specific content, design and language, VPs include also “cultural" features [6], which give a specific flavor and authenticity of the case. Those are difficult or impossible to translate. Moreover, VPs can be used for learning the background and the medical conditions of patients from different countries and cultures [6]. In our research we found many statistically significant differences in student activity through the cases, between those with Cluj authors (2010 - Cluj) and those developed in the USA (2011 - USA). These differences persist when we separately compared the Romanian and English versions of the two sets of VPs, so it can be deduced that they are not related to the language proficiency of students and are probably related to the “cultural" features of the cases, difficult or impossible to translate We found no statistical differences the ability of the students (2010 group versus 2011 group) to reach the correct diagnostic and therapeutic plan.

The increased mobility of healthcare professionals, students and patients, is intimately intertwined with medical education [29]. Many students are trained in countries other than where they were born. In addition, healthcare professionals often move between countries and are today meeting more and more patients from cultures different from their own [6]. There are important implications concerning the Internet and e-learning for globalization in medical education. Key components are a bank of reusable learning objects, a virtual practice with virtual patients, a learning-outcomes framework, and self-assessment instruments [30]. Cross-cultural development and international exchange of VPs could redress some of the imbalances between the developed world and the “vulnerable” countries [29].

## Conclusion

Virtual patient systems offer clinical skills training, clinical reasoning and decision making experiences that are impossible or impractical to gain elsewhere and in addition comprehensive and objective assessment. The development of a viable, financially self-sustaining VPs program in our University depends on the collaboration of a multi-institutional authoring process. Development of medical education programs with VPs in English would be feasible, cost-effective, and in accordance with the globalization in medical education.

## Competing interests

The authors declare that they have no competing interests.

## Authors’ contribution

VM was the principal author and contributed to the concept, design, literature search and interpretation of the data and drafting of manuscript. TC contributed with the acquisition of data and technical support. ST contributed to the analysis and interpretation of the data. UGHF provided the software used in the study, the Web-based Simulation of Patients (Web-SP) system, originating from the Karolinska Institutet, Stockholm, Sweden, contributed to the study concept and revision of the manuscript and provided important intellectual content. All authors read and approved the final manuscript.

## Authors' information

VM is the Assistant Dean for Education and an Associate Professor of General Surgery at the “Iuliu Hatieganu” University of Medicine and Pharmacy, Cluj-Napoca, Romania. He was a participant in the eViP Project and initiated the Virtual Patients Program at the Faculty of Medicine in Cluj-Napoca.

TC is an Associate Professor in Medical Informatics and Biostatistics Department at the “Iuliu Haţieganu” University of Medicine and Pharmacy, Cluj-Napoca, Romania. He was a member of the Technical Reference Group in the eVip Project and offers technical support for VPs applications.

ST is a Professor in Medical Informatics and Biostatistics Department at the “Iuliu Haţieganu” University of Medicine and Pharmacy, Cluj-Napoca, Romania. His recent research interests are focused on stochastic multiple criteria evaluation and ranking in medical field and applied biostatistics.

UGHF is Professor of IT and Learning at Stockholm University, Sweden.

## Pre-publication history

The pre-publication history for this paper can be accessed here:

http://www.biomedcentral.com/1472-6920/13/21/prepub
